# Chemical composition, antioxidant activity and in vitro antibacterial activity of *Achillea wilhelmsii* C. Koch essential oil on methicillin-susceptible and methicillin-resistant *Staphylococcus aureus* spp.

**DOI:** 10.1007/s13205-014-0197-x

**Published:** 2014-02-05

**Authors:** Seyedeh Mahsan Hoseini Alfatemi, Javad Sharifi Rad, Majid Sharifi Rad, Sasan Mohsenzadeh, Jaime A. Teixeira da Silva

**Affiliations:** 1 Department of Bacteriology and Virology, Shiraz Medical School, Shiraz University of Medical Sciences, P.O. Box 71455-119, Shiraz, Iran; 2 Zabol Medicinal Plants Research Center, Zabol University of Medical Sciences, P.O. Box 61615-585, Zabol, Iran; 3 Department of Pharmacognosy, Faculty of Pharmacy, Zabol University of Medical Sciences, P.O. Box 61615-585, Zabol, Iran; 4 Cereal Health Research Center of Zabol, Zabol University of Medical Sciences, P.O. Box 61615-585, Zabol, Iran; 5 Department of Range and Watershed Management, Faculty of Natural Resources, University of Zabol, Zabol, Iran; 6 Department of Rangeland Science, Gorgan University of Agricultural Sciences and Natural Resources, 49138-15739 Gorgan, Iran; 7 Department of Biology, College of Sciences, Shiraz University, 71454 Shiraz, Iran; 8 P.O. Box 7, Miki Cho Post Office, 3011-2, Ikenobe, Kagawa-Ken 761-0799 Japan

**Keywords:** *Achillea wilhelmsii*, Antioxidant activity, Essential oil (EO), GC–MS, MRSA, MSSA, *Staphylococcus aureus*

## Abstract

The present study investigated the chemical composition of the essential oil (EO) from aerial parts (flowering stage) of *Achillea wilhelmsii* C. Koch by GC–MS. In addition, the antioxidant activity of the EO as well as its antimicrobial activity against methicillin-susceptible and methicillin-resistant *Staphylococcus aureus* (MRSA) strains was tested. Antioxidant activity was measured by the ability of the EO to scavenge 1,1-diphenyl-2-picrylhydrazyl (DPPH) radicals while the antimicrobial activity was assessed by the disc-diffusion method. In total, 52 compounds were recognized, accounting for 97.33 % of the EO. The main compounds in the EO were carvacrol (22.49 %), dihydrocarvone (13.23 %), linalool (12 %), 1,8-cineol (11.42 %), camphene (8.31 %), thymol (5.28 %), camphor (3.71 %), pulegone (2.82 %) α-terpineol (2.11 %), bornyl acetate (1.14 %), and farganol (1.01 %). The EC_50_ value of the EO was 0.01 and 0.08 mg/mL for the antioxidant and DPPH-scavenging ability, respectively. *A. wilhelmsii* EO affected methicillin-sensitive *Staphylococcus aureus* (MSSA) and MRSA, but the impact was more effective on MSSA.

## Introduction

Herbal medicines are considered to be an important natural medicine for the treatment of health conditions and diseases. The excessive and repeated use of the same drugs used in modern medicine has led to the evolution of antibiotic-resistant microbes, including *Staphylococcus aureus* whose emergence of antibiotic-resistant strains reduces the number of antibiotics available to treat clinical infections caused by this bacterium (Parker and Jevons 1964). *S. aureus* is a highly versatile pathogen with considerable importance in human medicine. *S. aureus* is responsible for a wide range of hospital and community-acquired infections worldwide, from skin infections and food poisoning to life-threatening situations such as toxic-shock syndrome, endocarditis, pneumonia, bacteraemia and osteomyelitis (Kim et al. 2006; Akineden et al. 2008). Traditional medicine involving herbs (or the compounds within them) can solve health and medical problems caused by *S. aureus*. The essential oils (EOs) (primarily from leaves) of *Thymus vulgari*s and *Eucalyptus globulus* were tested against clinical isolates of methicillin-resistant *Staphylococcus aureus* (MRSA), both EOs to possessing antibacterial activity against MRSA, the former being more potent than the latter (Tohidpour et al. 2010). *Daucus crinitus* (a medicinal plant) EOs (derived from stems and leaves of wild plants) inhibited *S. aureus* (Bendiabdellah et al. 2013).

*Achillea wilhelmsii* C. Koch a perennial medicinal herb belonging to the Asteraceae family has a relatively wide distribution in different parts of Iran (Rechinger 1963; Mozaffarian 1966). It is native to Western Asia and Europe, although populations have also been discovered in North America, Australia and New Zealand (Dokhani et al. 2005). *A. wilhelmsii* has a wide range of reported biological activities, including antispasmodic (Yaeesh et al. 2006), antacid (Niazmand et al. 2010), antioxidant (Candan et al. 2003; Baris et al. 2006; Nemeth and Bernath 2008; Fathi et al. 2011), antihyperlipidemia (Asgary et al. 2000), antihypertensive (Niazmand et al. 2011) and antitumoral (Csupor-Loffler et al. 2009).

The hygiene industry utilizes *A. wilhelmsii* EO to make skin tender and soft and to treat skin inflammations using cream formulations (Pieroni et al. 2004). *A. wilhelmsii* is rich in sesquiterpenes, lactones flavonoids and monoterpenoids which have antioxidant activities (Jaimand and Rezaee 2001; Saeidinia et al. 2005).

The main purpose of the present study was to perform a biological examination on *A. wilhelmsii* C. Koch from Golmakan Khorasan Razavi, Iran by assessing the antioxidant and antimicrobial activity of the EO.

## Materials and methods

### Plant preparation and procedure

**Fig. 1:**
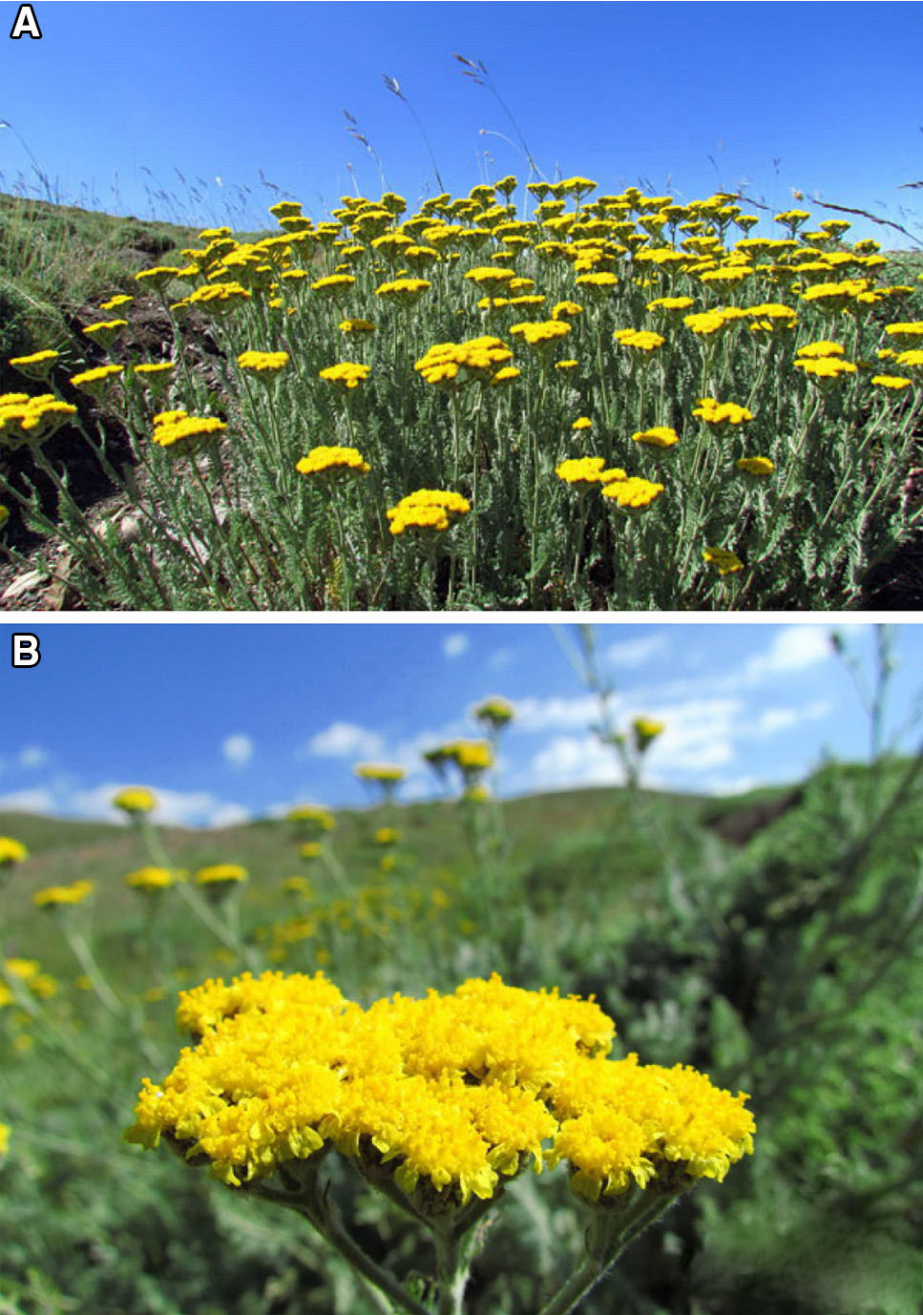
*A. wilhelmsii* C. Koch in the flowering stage used for EO analyses in this study. **a** Wild population; **b** close-up of flowering capitulum

Aerial parts (stems, leaves and flowers) of the flowering stage of *A. wilhelmsii* C. Koch (Fig. 1a, b) were collected in June 2012 from Golmakan (36°28′44″N, 59°9′17″E), Khorasan Razavi, Iran. The plant was taxonomically identified by a botanist at the herbarium of Pharmacognosy, Department of the Faculty of Pharmacy affiliated to Shahid Beheshti University of Medical Sciences of Iran.

### Extraction and isolation of the EO

Plant parts were air-dried in the shade at ambient temperature (18–25 °C) for 12 days. Dried aerial parts (100 g) were cut into small pieces and hydro-distilled for 4 h using Clevenger-type apparatus. The resulting EO was dried over anhydrous sodium sulfate and stored at 4 °C until GC–MS analysis and bioassays.

### GC–MS analysis

In this study, an HP 6890 N GC system coupled with an HP MSD5973 N quadruple mass spectrometer was utilized. The extracted compounds were separated on an HP-5MS capillary column (30 m length, 0.25 mm internal diameter, 0.25 mm film thickness). Split injection:sample ratio for distillation was 50:1. The column oven temperature was programmed to rise from an initial 40 to 150 °C at 4 °C/min, and then to 240 °C at 10 °C/min. Injection temperature and ion source temperature were 240 °C. Helium was used as the carrier gas with a flow rate of 1.2 mL/min. The ionizing energy was 70 eV. All data were obtained by collecting the full-scan mass spectra within the scan range 50–550 amu. Compounds were identified using the Wiley 7n.L Mass Spectral Library (Wiley, New York, NY, USA). Trace compounds were defined as those detected at <0.04 % of the EO.

### Antioxidant activity

Antioxidant activity was measured by the paired diene method (Lingnert et al. 1979). The antioxidant activity measured is the ability of the EO to inhibit the peroxidation of linoleic acid in which the double bond is altered to a paired diene. Each EO sample (0.01–30 mg/mL) in methanol (100 μL) was mixed with 3 mL of 10 mM linoleic acid (Sigma Chemical Co., St. Louis, MO, USA) to form an emulsion in 0.2 M sodium phosphate buffer (pH 6.6) in test tubes and placed in the dark at 37 °C to quicken oxidation. After incubation for 17 h, 7 mL of 70 % methanol in deionized water was added, and the absorbance of the mixture was measured at 234 nm against a blank in a Hitachi U-2001 spectrophotometer (Tokyo, Japan). Antioxidant activity was quantified as follows: Antioxidant activity (%) = [(∆*A*_234_ of control − ∆*A*_234_ of sample)/∆*A*_234_ of control] × 100. Analyses were repeated three times. α-Tocopherol, butylated hydroxyanisole (BHA) and ascorbic acid (Sigma) were used as standard controls.

### Scavenging ability on 1, 1-diphenyl-2-picrylhydrazyl radicals

The scavenging ability of 1,1-diphenyl-2-picrylhydrazyl (DPPH, Sigma) radicals, which was measured by the method of Shimada et al. (1992), is the ability of the EO to react quickly with DPPH radicals and to decrease most DPPH radical molecules. The assay was repeated three times. α-Tocopherol, BHA and ascorbic acid were used as standard controls. Each EO sample (0.5–30 mg/mL) in methanol (5 mL) was mixed with 1 mL of methanolic solution containing DPPH radicals, resulting in a final concentration of 0.2 mM DPPH. The mixture was shaken vigorously, left to stand for 45 min in the dark, and the absorbance was then measured at 517 nm against a blank. The scavenging ability was calculated as follows:

Scavenging ability (%) = [(∆*A*_517_ of control − ∆*A*_517_ of sample)/∆*A*_517_ of control] × 100.

EC_50_ value (mg/mL) is the efficient concentration at which the antioxidant activity was inhibited by 50 % and DPPH radicals were scavenged by 50 %, and was gained by interpolation from linear regression analysis.

### Antibacterial study

#### Preparation of microorganisms

The *S. aureus* strains utilized in this study were clinical isolates from patients with *S. aureus*, obtained from the microbiological laboratory of the central hospital in Shiraz, Iran. This study was approved by the ethics committees of Zabol and Shiraz Universities of Medical Sciences. MRSA that were isolated were identified by screening tests on Mueller–Hinton agar (MHA, Torlak, Berlin, Germany) supplemented with 5 % NaCl and 1 mg/mL oxacillin-impregnated disc to isolate MRSA (Roberts et al. 2002). Finally, 10 MRSA strains and 5 methicillin-susceptible *S. aureus* (MSSA) strains were isolated from patients. In this study, two standards strains, ATTC 25923 (MRSA) and PTCC 1341 (MSSA), were used.

#### Disc-diffusion assay

Antimicrobial tests were performed by the disc-diffusion method using 100 μL of suspension (containing 2.0 × 10^8^ CFU/mL of bacteria) dispersed evenly on MHA in sterilized Petri dishes (80 mm in diameter). To the discs (6 mm in diameter, HiMedia Laboratories Pvt. Ltd., Mumbai, India), 20, 50, 100 and 200 μL of EO and placed on the inoculated agar. The inoculated plates were maintained at 4 °C for 2 h and incubated at 37 °C for 24 h. Antimicrobial activity was evaluated by measuring the zone of inhibition (mm) against the test bacterial (MRSA and MSSA) strains.

### Statistical analysis

The EO was prepared in triplicate for chemical characterization and for antioxidant and antibacterial assays. Data was subjected to analysis of variance following a completely random design to determine the least significant difference (LSD) at *P* < 0.05 using SPSS v. 11.5.

## Results and discussion

### The composition of *A. wilhelmsii* essential oil

The mass spectra and retention indices (RI) were used in this study to determine the EO composition of *A. wilhelmsii*. In total, 52 compounds were identified accounting for 97.33 % of the EO components (Table 1). The main compounds of the EO were (in decreasing order) carvacrol (22.49 %), dihydrocarvone (13.23 %), linalool (12 %), 1,8-cineol (11.42 %), camphene (8.31 %), thymol (5.28 %), camphor (3.71 %), pulegone (2.82 %), α-pinene (2.2 %), terpineol (2.11 %), bornyl acetate (1.14 %) and farganol (1.01 %). Some compounds were detected in trace amounts (not listed in Table 1): heptanal, isopentyl isovalerate, neo-3-thujanol, *cis*-jasmone, elemol. The main compound in the EO of *A. wilhelmsii* from the Golmakan Khorasan Razavi (Iran) area was carvacrol (22.42 %). Javidnia et al. (2004) also found 25.1 % carvacrol in *A. wilhelmsii* oil as the main compound. The major constituent of the EO of the flowers and leaves of *A. wilhelmsii* from Mazandaran (Iran) province was camphor, 21.2 % and 24.1 %, respectively (Azadbakht et al. 2003). Carvacrol and camphor have no harmful effects on humans and environment (Rajendran and Sriranjini 2008; Khani and Asgari 2012). The amount of camphor in *A. wilhelmsii* EO collected from Kerman was 9.0 % (Afsharypuor et al. 1996) and in the EO of aerial parts from Kazeroon (Iran, Fars) province was 2.2 % (Javidnia et al. 2004). Afsharypuor et al. (1996) reported that main compound in the EO of *A. wilhelmsii* was caryophyllene oxide (12.5 %), much higher than that reported in our study (0.08 %). 1,8-cineol, which was found at 3.32 % of the stem EO in this study was the major constituent of the oil of *A. wilhelmsii* from Egypt and Turkey (Javidnia et al. 2004; Baris et al. 2006). The variations in the qualitative and quantitative composition of the EOs from different locations within the same country or from different countries are likely caused by genetic variation, growth conditions, geographic variation and analytical protocols used to assess the EOs. Previous studies showed that monoterpenes, the main part of the *A. wilhelmsii* EO from Golmakan, have influential insecticidal effects against stored product insects (Papachristos et al. 2004; Rajendran and Sriranjini 2008). Consequently, the *A. wilhelmsii* EO from Golmakan could be an important optional phytochemical control strategy without undesirable effects such as direct toxicity to humans and environmental pollution (Rajendran and Sriranjini 2008; Khani and Asgari 2012). In addition, *A. wilhelmsii* EO contains sesquiterpenes, lactones and flavonoids, which have in the ability to lower blood lipid levels and hypertension (Asgary et al. 2000).

**Table 1:** Composition of the EO of *A. wilhelmsii*, identified by RI-MS*, relative to the literature

No.	Name of compound	RI	Relative % in EO
1	2-Methyl-butyl-acetate	885	0.43
2	Heptanal	910	0.01
3	*α*-Thujene	926	0.81
4	*α*-Pinene	935	2.22
5	Camphene	952	8.31
6	Sabinene	978	0.64
7	*β*-Pinene	984	0.78
8	3-Octanone	989	0.32
9	*α*-Phellandrene	1010	0.27
10	*α*-Terpinene	1015	0.73
11	*p*-Cymene	1028	0.81
12	1,8-Cineol	1034	11.42
13	Phenylacetaldehyde	1039	0.18
14	*γ*-Terpinene	1056	0.64
15	*cis*-Sabinene-hydrate	1064	0.16
16	*trans*-Linalool oxide	1076	0.29
17	Terpinolene	1086	0.31
18	*α*-Pinene oxide	1094	0.19
19	Linalool	1105	12.0
20	*cis*-Thujone	1108	0.21
21	Hotrienol	1111	0.25
22	Isopentyl isovalerate	1113	0.03
23	*α*-Thujone	1119	0.12
24	α-Campholenal	1123	0.32
25	Allo-Ocimene	1129	0.12
26	Isopinocarveol	1130	0.11
27	*trans*-Sabinol	1137	0.24
28	Camphor	1140	3.71
29	*p*-Menth-3-en-8-ol	1144	0.4
30	*neo*-3-Thujanol	1149	0.01
31	*trans*-*β*-Terpineol	1158	0.38
32	Borneol	1162	0.42
33	*n*-Nonanol	1166	0.01
34	4-Terpineneol	1170	0.37
35	*para*-Methyl-acetophenone	1175	0.12
36	*para*-Cymen-8-ol	1180	0.14
37	*α*-Terpineol	1185	2.11
38	Myrtenol	1190	0.21
39	Verbenone	1204	0.26
40	Farganol	1209	1.01
41	*trans*-Carveol	1218	0.12
42	Dihydrocarvone	1228	13.23
43	Pulegone	1248	2.82
44	Bornyl acetate	1252	1.14
45	Thymol	1288	5.28
46	Carvacrol	1328	22.49
47	Verbanol acetate	1369	0.25
48	*cis*-Jasmone	1398	0.04
49	Neryl isobutyrate	1467	0.38
50	Menthyl isovalerate	1532	0.42
51	Caryophyllene oxide	1549	0.08
52	Elemol	1568	0.01

*RI* retention indices relative to C6–C24 *n*-alkanes on the DB-5 column; *MS* mass spectrum (as indicated by the Wiley 7n.L Mass Spectral Library)

### Antioxidant activity and scavenging ability

The results for antioxidant activity and scavenging ability on DPPH radicals of the EOs assayed are summarized in Table 2. The efficiency of antioxidant activity and scavenging ability is inversely related with their EC_50_ values. The antioxidant activity EC_50_ values were 0.08, 0.05, 4.07 and 0.01 mg/mL for α-tocopherol, BHA, ascorbic acid and *A. wilhelmsii* EO, respectively. The scavenging ability EC_50_ values was 0.11, 0.07, 9.09 and 0.58 mg/mL for α-tocopherol, BHA, ascorbic acid and *A. wilhelmsii* EO, respectively. The antioxidant activity of *A. wilhelmsii* EO was stronger than three standards tested (Table 2), which could be used for the treatment of human diseases to remove free radicals (Dharmendra et al. 2009).

**Table 2:** EC_50_ values (mg/mL) of the *A. wilhelmsii* EO in two assays

	Antioxidant activity (mg/mL)	Scavenging ability (mg/mL)
*A. wilhelmsii* EO	0.01 ± 0.02 d	0.08 ± 0.08 d
α-Tocopherol	0.08 ± 0.01 b	0.11 ± 0.01 b
BHA	0.05 ± 0.01 c	0.07 ± 0.01 c
Ascorbic acid	4.03 ± 0.07 a	9.09 ± 0.06 a

Values are mean of ±SD of three replicates. Mean values with different letters within a column are significantly different (*P* < 0.05; LSD)

### Antibacterial activity

The results of antibacterial activity of EOs (Table 3) showed that the maximum level of EO (200 μL) was inhibitory (largest zone of inhibition) against MSSA (22.56 mm) and MRSA (14.22 mm). The inhibitory activity against MSSA was greater than against MRSA. The EOs of *A. wilhelmsii* had a more negative impact on MSSA than MRSA (Table 3). Monoterpenes, which are rich in the EO of *A. wilhelmsii*, have powerful antibacterial effects (Unlu et al. 2002; Sokmen et al. 2004; Prabuseenivasan et al. 2006). In addition, phenolic and flavonoid compounds, also present in the EO of *A. wilhelmsii*, have antimicrobial activity (Stojanovic et al. 2005; Eleyinmi 2007; Yaghoubi et al. 2007; Mothana et al. 2009).

**Table 3:** Antibacterial activity of the EO of plants against MSSA standard, MSSA (*n* = 5), MRSA standard, and MRSA (*n* = 10) strains Diameter of the zone of inhibition (mm)

EO volume (μl)	MSSA standard	MSSA (*n* = 5)	MRSA standard	MRSA (*n* = 10)
25	14.01 ± 1.01	11.56 ± 1.04	6.25 ± 0.38	7.55 ± 0.28
50	16.01 ± 0.01	15.84 ± 0.89	12.1 ± 0.28	9.00 ± 0.33
100	14 ± 1.38	18.49 ± 1.23	12 ± 1.01	12.46 ± 1.07
200	27 ± 0.079	22.56 ± 0.45	19 ± 0.017	14.22 ± 0.11

Values are mean of ±SD of three replicates

## Conclusion

In this study, chemical composition, antioxidant activity and in vitro antibacterial activity of *A. wilhelmsii* L. Essential oil on methicillin-susceptible and methicillin-resistant *S. aureus* spp. was investigated. *A. wilhelmsii* C. Koch has emerged as an important medicinal plant. Its EO could be commercialized for its antioxidant, insecticidal and antibacterial applications, or used in the pharmaceutical, cosmetic or perfume industries. 

## Abbreviations

*A. wilhelmsii*: *Achillea wilhelmsii*
BHA: Butylated hydroxyanisole
EO: Essential oil
DPPH 1: 1-Diphenyl-2-picrylhydrazyl
GC–MS: Gas chromatography–mass spectrometry
MRSA: Methicillin-resistant *Staphylococcus aureus*
MSSA: Methicillin-sensitive *Staphylococcus aureus*
*S. aureus*: *Staphylococcus aureus*
